# BP Network Model Based on SCLBOA for House Price Forecasting

**DOI:** 10.1155/2022/8148586

**Published:** 2022-10-13

**Authors:** Xudong Ji, Xiao-Fang Ji, Hongxing Wei, Youdong Chen, Wei Xue

**Affiliations:** School of mechanical engineering and automation, Beihang University, Beijing 100191, China

## Abstract

Butterfly optimization algorithm (BOA) is a new swarm intelligence algorithm mimicking the behaviors of butterflies. However, there is still much room for improvement. In order to enhance the convergence speed and accuracy of the BOA, we present an improved algorithm SCLBOA based on SIBOA, which incorporates a logical mapping and a Lévy flight mechanism. The logical chaotic map is used for population initialization, and then the Lévy flight mechanism is integrated into the SCLBOA algorithm. To evaluate the performance of the SCLBOA, we conducted many experiments on standard test functions. The simulation results suggest that the SCLBOA is capable of high-precision optimization, fast convergence, and effective global optimization, all of which show that our method outperforms other methods in solving mathematical optimization problems. Finally, the BP network is optimized according to the SCLBOA (SCLBOA-BP) to further verify the availability of the algorithm. Simulation experiments prove the practicability of this method by building a Boston housing price prediction model for training.

## 1. Introduction

Inspired by human intelligence, the social behaviors of biological groups, or the laws of nature, many intelligence optimization algorithms are developed to solve complex optimization problems, which represents the applications of artificial intelligence. As a subfield of artificial intelligence, swarm intelligence requires effective computational methods, as the algorithms themselves must show high levels of adaptability to complex and constantly changing situations to find optimal solutions. The metaheuristic methods for the optimization problem are proved to be a good solution [1].

General purposed metaheuristic methods are evaluated in eight different groups which are biology based, swarm based, math based, sport based, chemistry based, social based, music based, and physics based. Furthermore, there are hybrid methods that are a combination of these [1]. Genetic algorithm (GA) which solves both constrained and unconstrained optimization problems that are based on natural selection [2, 3], differential evolution (DE) which optimizes a problem by iteratively improving a candidate solution based on an evolutionary process [4], and slime mould algorithm (SMA) which is an effective optimizer motivated by slime behavior to tackle the optimization problems [5] are biology based; particle swarm optimization (PSO) which optimizes a problem by iteratively trying to improve a candidate solution with regard to a given measure of quality [6], cat swarm optimization (CSO) which is inspired by resting and tracing behaviors of cats [7, 8], grey wolf optimization (GWO) which simulates the leadership hierarchy and hunting mechanism of grey wolves in nature [9], and Harris hawks optimization (HHO) which is a gradient-free optimization algorithm with several active and time-varying phases of exploration and exploitation [10] are swarm based; sine cosine algorithm (SCA) which generates various initial random solutions and asks them to shift towards the best solution using a mathematical model [11] is math based; war strategy optimization (WSO) which is based on the strategic movement of army troops during the war is sport based [12]; artificial chemical reaction optimization algorithm (ACROA) which mimics chemical reaction process is chemistry based [13]; teaching-learning-based optimization (TLBO) which is based on the effect of the influence of a teacher on the output of learners in a class is social based [14]; harmony search algorithm (HS) which is inspired by music to solve computationally involved optimization paradigms is music based [15]. Chaos optimization algorithm (COA) which usually utilizes the chaotic map like a logistic map to generate the pseudo-random numbers mapped as the design variables for global optimization can be classified as both math based and physics based [16, 17].

These primitive intelligent algorithms are simple and easy to implement, with fewer parameters and shorter running time. Therefore, it presents excellent operability and optimization ability in solving many nonlinear and multimodal realistic optimization problems. BOA [18] was proposed by Arora and Singh in 2018. The method and concept of this algorithm were first proposed at the 2015 International Signal Processing, Computing, and Control Conference (2015 ISPCC) [19]. After the algorithm was proposed, the authors conducted a lot of research on BOA [20, 21]. In the BOA algorithm, each butterfly has its own unique sense and individual perception ability. This is also a major feature that differentiates it from other metaheuristics.

As a newly proposed natural heuristic algorithm, BOA, like other intelligent algorithms, also has defects such as convergence speed, and it is easy to fall into local optimum. In response to the above problems, many scholars have proposed different improvement strategies. Wang and Zhang [22] introduced adaptive inertial weights and introduced multisegment perturbation strategies into the update of the optimal nectar position, introduced the crazy factor into the position formula to increase the population diversity, and proposed a crazy butterfly algorithm based on adaptive perturbation (CIBOA). Gao and Liu [23] first introduced the limit threshold to limit the number of times BOA fell into the local optimal solution and combined the simplex strategy to optimize the poorly positioned butterflies at the later stage of the iteration to improve the performance of the algorithm. BOA is a bionic swarm intelligence algorithm derived from simulating the foraging or courtship behavior of butterflies in nature. It has successfully solved engineering design problems [24], image segmentation [25], and data mining [26]. But these optimizations are difficult to balance the system's computational power and exploration power.

The SCLBOA algorithm differs from the traditional BOA algorithm in that it is developed based on the SIBOA algorithm with an integration of the logical chaotic map and the Lévy flight mechanism. This paper aims to balance the exploratory capacity and exploitative capability of the SCLBOA. The major contributions of this study are summarized as follows:

- The method used for initializing the individuals of the population is a chaotic sequence generated based on logistic mapping, which exhibits higher uniformity and diversity and helps to improve the efficiency and quality of the solution.
- The Lévy flight mechanism is introduced in the solution search to generate random steps. Randomly smaller steps for long-distance and more giant steps for short-distance are applied to prevent the solution search from stagnating.
- To balance the local search ability and global search ability of the algorithm, individuals' Lévy flight state is adjusted periodically, and a limit range is set, both of which are realized by changing the mathematical function.

The rest of the paper is organized as follows.  Section 2 introduces related work and existing BOA and SIBOA algorithms.  Section 3 proposes a new SCLBOA algorithm and introduces the improved algorithm in detail.  Section 4 explains the solution quality and convergence performance of benchmark functions.  Section 5 uses SCLBOA as the training algorithm for the BP network, that is, the SCLBOA-BP network predicts Boston housing prices.  Section 6 provides an in-depth analysis of the method proposed in this paper.  Section 7 depicts the main works in this study and gives some suggestions for future research.

## 2. Related Research Works

### 2.1. BOA

BOA [18, 19] is a metaheuristic algorithm inspired by natural organisms, which simulates the foraging and mating behavior of butterflies. In order to determine the potential direction of mating objects and food sources, butterflies judge by a certain concentration of aroma in the air. The scent intensity is determined by each butterfly and directly determines the fitness of the butterfly to find the best, so the fitness of the butterfly will vary according to the change of position. The global search in BOA is that the butterflies in the population move towards the target individual that emits fragrance, and the local search is the random movement of butterflies when they do not perceive the fragrance of other individuals. The concentration of the butterfly's fragrance depends on the following three factors: sensory factor, stimulation intensity, and power index. The equation is as follows:

(1) $$\begin{array}{c} f=cI^{a}, \end{array}$$

where *f* is the scent concentration, *c* is the sensory factor, usually taking the value of 0.01, *a* is a power exponent that depends on *f*, usually taking the value of 0.1, and *I* is the stimulus intensity, which is related to the optimal fitness.

Before BOA begins a local search and global search, the algorithm will randomly generate the positions of the population individuals and generate their respective fragrances accordingly.

In the common global search stage, when a butterfly senses the smell of other butterflies, it moves towards the current global optimal position *g*_best_. *x*_*i*_^*t*^ is the position vector of the *i*-th butterfly in the *t*-th iteration, which is the part of its own cognitive flight; *f*_*i*_ is the scent size of the *i*-th butterfly; *r*_1_ is a random number in [0,1].

(2) $$\begin{array}{c} x_{i}^{t+1}=x_{i}^{t}+f_{i}\left(r_{1}^{2}g_{\text{best}}-x_{i}^{t}\right). \end{array}$$

In another case when a butterfly cannot sense the smell of its nearby environment, it moves randomly. Intensive local search updates as equation (3). Among them, *x*_*j*_^*t*^, *x*_*k*_^*t*^ are the *j*-th butterfly and the *k*-th butterfly randomly selected from the solution space; *r*_2_ is a random number in [0,1].

(3) $$\begin{array}{c} x_{i}^{t+1}=x_{i}^{t}+f_{i}\left(r_{2}^{2}x_{j}^{t}-x_{k}^{t}\right). \end{array}$$

In the butterfly foraging process, the switching probability *P*=0.8 controls whether the algorithm is in the dense local search or the ordinary global search stage. Each iteration compares the switch probability *P* and the size of a random number *r*_3_ to decide whether to perform a global search or a local search. The final update formula of the butterfly algorithm is as follows:

(4) $$\begin{array}{c} x_{i}^{t+1}=\left\{\begin{array}{c} x_{i}^{t+1}=x_{i}^{t}+f_{i}\left(r_{1}^{2}g_{\text{best}}-x_{i}^{t}\right),r_{3}<P,\\ x_{i}^{t+1}=x_{i}^{t}+f_{i}\left(r_{2}^{2}x_{j}^{t}-x_{k}^{t}\right),r_{3}\geq P. \end{array}\right. \end{array}$$

### 2.2. SIBOA

In the butterfly optimization algorithm, the position of the nectar source of the population plays an important role as it guides the individual butterfly to move to the optimal solution. However, if the population falls into the local optimal position, it will easily cause the search to stop in the group, and a better global optimization cannot be obtained. Therefore, in order to balance the ability of local search and global search of BOA, SIBOA [27] introduces the sine cosine operator in its own cognitive flight part:

(5) $$\begin{array}{c} x_{i}^{t+1}=\left\{\begin{array}{c} x_{i}^{t+1}=x_{i}^{t}r_{4}\sin r_{5}+f_{i}\left(r_{1}^{2}g_{\text{bes}t}-x_{i}^{t}\right),r_{3}<P,\\ x_{i}^{t+1}=x_{i}^{t}r_{4}\cos r_{5}+f_{i}\left(r_{2}^{2}x_{j}^{t}-x_{k}^{t}\right),r_{3}\geq P, \end{array}\right.\\ r_{4}=2-2\left(\frac{t}{{\text{Max}}_{\text{iter}}}\right),\\ r_{5}=2\pi \cdot \text{rand}, \end{array}$$

where *t* represents the current number of iterations; *Max_iter* represents the maximum number of iterations; *r*_1_, *r*_2_, *r*_3_ represent a random number within [0, 1]; *P* is the switching probability, which determines the choice of algorithm update formula; *r*_4_ is the control parameter, which determines the location area of the *i*-th butterfly individual in the next iteration; *r*_5_ is a random number in [0,2*π*], which defines whether the current solution should be close to the target or far away from the target; and *r*_4_sin*r*_5_ and *r*_4_cos*r*_5_ jointly control the algorithm for local search and global search. When the value of *r*_4_sin*r*_5_ and *r*_4_cos*r*_5_ is greater than 1 or less than −1, develop different areas for global search. When it is between −1 and 1, develop the desired search space for local search.

In SIBOA, the scent size changes with the degree of butterfly absorption, which is realized by the power index parameter *a*. The behavior of the individual butterfly in the control algorithm affects the convergence speed and optimization accuracy of the entire algorithm. When *a* = 1, it means that no fragrance is absorbed, which means that the fragrance is spread in an ideal environment. The fragrance emitted by the butterfly can be felt from anywhere in the search domain, and the global optimal value can be easily obtained. *a* = 0 means that the fragrance of any butterfly cannot be detected by other butterflies. Therefore, in the original BOA, the power exponent *a* that depends on the scent size *f* is set to a specific value of 0.1, which results in poor optimization performance. Therefore, *a* in SIBOA adopts the following calculation formula:

(6) $$\begin{array}{c} a=a_{\text{max}}\left(a_{\text{max}}-a_{\text{min}}\right)\left(\frac{\text{Max}\_\text{iter}-t}{\text{Max}\_\text{iter}}\right), \end{array}$$

where *a*_max_, *a*_min_ represent the maximum and minimum power exponent coefficients, respectively. The value of *a* decreases linearly from 0.3 to 0.01, which can effectively adjust the butterfly's absorption of fragrance, which is convenient for the individual butterfly to perform better local search and global search and improves the accuracy of convergence.

## 3. Fusion of Logistic Chaotic Mapping and Lévy Flight Sine-Cosine Butterfly Optimization Algorithm (SCLBOA)

### 3.1. Logistic Chaotic Map Initialization

Studies have shown that the initialization of the population has a certain impact on the performance of the algorithm. As the chaotic sequence can be easily generated, it has attracted significant attention from researchers. Recently, chaotic ensemble optimization has been adopted in many studies[28, 29]. Richards et al. were the earliest ones who proposed such insights [30]. A slight difference in the initial state of a nonlinear system will lead to very different state development and change. The development and change of the position of the population are chaotic, and different initial positions will produce very different solutions. If the initial population is better, it will improve the solution efficiency and the quality of the solution, so the population individuals should be uniformly initialized in the solution space as much as possible.

The initialization process of the basic BOA is random. However, based on the sine operator introduced in SIBOA, the improvement of SCLBOA uses the logistic mapping in the chaotic sequence to initialize the position and velocity of the butterfly due to the chaotic sequence. Those with nonrepetitive ergodicity and pseudo-random characteristics can help increase the diversity of the population.

Logistic mapping is currently the most widely used nonlinear dynamics discrete chaotic mapping system. Its mathematical model is defined as follows:

(7) $$\begin{array}{c} x_{i+1}=S\left(x_{i}\right)=\alpha x_{i}\left(1-x_{i}\right),\;\left\{i=1,2,3,\cdots \right\}, \end{array}$$

where *x*_*i*_ is the input and *x*_*i*+1_ is the output, both of which are the state values of the logistics mapping; *α* represents system control parameters; and *S*(*x*_*i*_) is the logistic mapping between variable *x*_*i*_ and control parameter *α*. Logistic mapping is used to generate chaotic variables to describe position and velocity. At this time, the chaotic position variables generated have better ergodicity.

**Figure 1:**
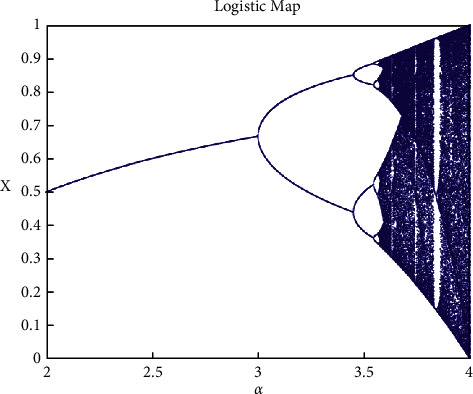
Logistic map bifurcation.

Figure 1 shows the bifurcation diagram of logistic mapping.

When x takes a value between [0, 1] and α takes a value between [2, 4], the logistic map bifurcation graph is shown above. When α=3.569945672, it enters the chaotic state.

### 3.2. Lévy Flight Mechanism

The SCLBOA proposed in this paper not only uses the logistics chaotic map but also introduces the Lévy flight mechanism [31, 32]. The Lévy flight search strategy is mainly used to generate random steps, similar to a random walk, where the solution search moves towards a completely random direction at every step.

The characteristic performance of the Lévy flight mechanism is similar to the global search and local search features in the intelligent optimization algorithm. In one case, it repeatedly walks randomly with a small step size over a long distance to ensure that it can enter another area and search in a wider range. In another case, the direction mutation jump is occasionally performed in a short distance with a large step size, to ensure that the individual carefully searches the small area around itself. Its step size satisfies the form of power-law distribution, which can be expressed as follows:

(8) $$\begin{array}{c} Levy\left(x\right)=0.01\frac{r\sigma }{\left|r^{\left(1/\xi \right)}\right|}, \end{array}$$

(9) $$\begin{array}{c} \sigma =\left(\frac{\Gamma \left(1+\xi \right)\cdot \sin\left(\pi \xi /2\right)}{\Gamma \left(\left(1+\xi \right)/2\cdot \xi \cdot 2^{\left(\left(\xi -1\right)/2\right)}\right)}\right)\;where\;\Gamma \left(x\right)=\left(x-1\right)!, \end{array}$$

where *r* is a random number in the range of [0, 1] and the value of *ξ* is set to 1.5.

In this paper, the experiment sets the limit range. When it is less than 1/5 of the population, the Lévy flight strategy is introduced to improve the global search ability; when it is greater than 1/5 of the population, the butterfly updates its position compared with the worst value. The core idea is to change the individual state of the butterfly population through the change of mathematical functions, increase the diversity of the population, and improve the global search ability. The updated position formula after introducing Lévy flight is as follows:

(10) $$\begin{array}{c} x_{i}^{t+1}=\left\{\begin{array}{c} Q\cdot \exp\left(\frac{\left(x_{\text{worst}}^{t}-x_{i}^{t}\right)}{i^{2}}\right),\frac{i>\text{popsize}}{5},\\ x_{p\text{best}}^{t}+x_{p\text{best}}^{t}⊗Levy\left(\text{dim}\right),\text{ other}. \end{array}\right. \end{array}$$

### 3.3. Algorithm Steps

The specific steps of SCLBOA are as follows:

 
*Step 1*. Initialize parameters. Use logistic mapping to chaotically initialize the population position within the boundary range to ensure that the initial position traversal is distributed in the search space.
 
*Step 2*. Calculate the initial fitness value. Calculate the fitness value of the individual butterfly according to the test function.
 
*Step 3*. Select the source of nectar. The butterfly position with the best fitness value is selected as the nectar source position, and the fragrance size is calculated according to formula (1).
 
*Step 4*. Update the butterfly position. In order to reduce the influence of external environmental factors, according to (2)formulas –(4) and the random number P, it is judged whether the current iteration is performing a global search or a local search. The position update formulas (5) and (8)–(10) use the sine cosine operator and the Lévy flight mechanism.
 
*Step 5*. Calculate the fitness value and update the optimal position. Compare the target value of the current individual of the butterfly with the previous individual and replace it if it is superior.
 
*Step 6*. Repeat the iterative process of Step 4 and Step 5. If the set accuracy requirements or the specified maximum number of iterations are reached, the algorithm is terminated and the global optimal solution is output.

### 3.4. SCLBOA Flowchart

**Figure 2:**
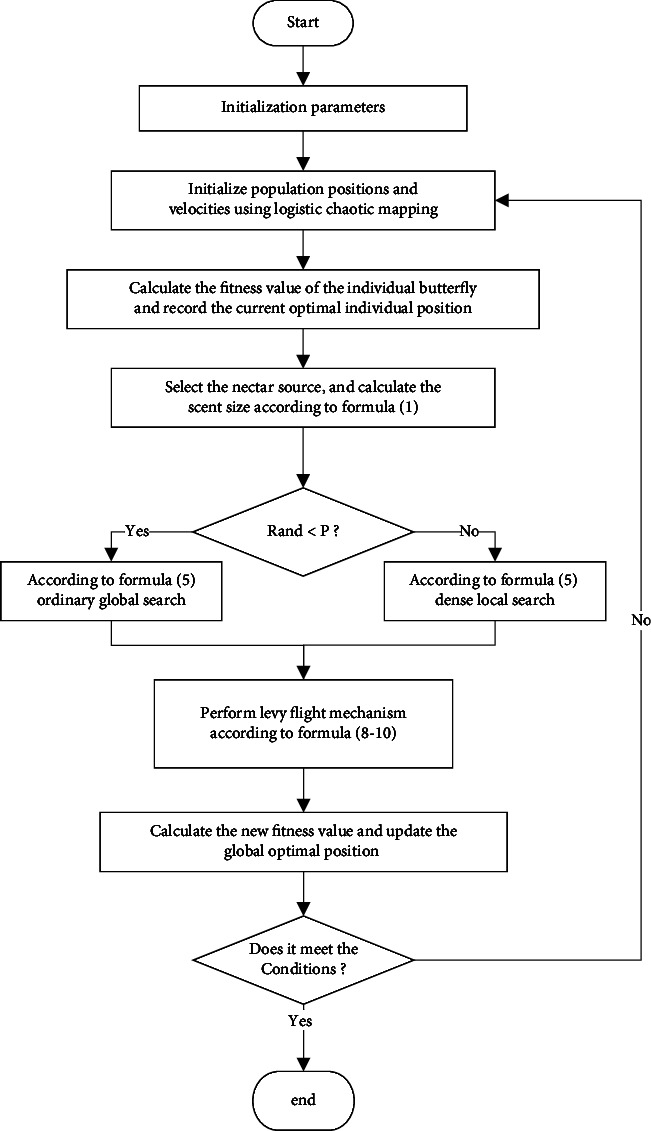
SCLBOA algorithm flowchart.

According to the description of SIBOA improvement in Section 3, the specific implementation process of SCLBOA is shown in Figure 2.

When running the SCLBOA algorithm, the initialization is performed first, and then the iterative search is performed, and in the final phase, the algorithm running terminates until the optimal solution is found. In the initialization stage, by initializing the position of the population through chaotic logic mapping, the algorithm defines the target function and its solution space and records the optimal position of individuals while calculating the individual fitness value. At the multiple-iteration stage, individual butterflies in the solution space move and update their positions following the Lévy flight mechanism, thereby evaluating their fitness values. When the maximum number of iterations is completed, the iteration ends, and the algorithm outputs the optimal solution with the highest fitness value. The above steps constitute the whole procedure of the SCLBOA algorithm.

## 4. Experimental and Result Analysis

In this section, to find the optimal solution, the exploratory and exploitative capacity of the proposed method is examined by eight typical standard test functions in different dimensions. Also, six algorithms (i.e., PSO, DE, SCA, BOA, SIBOA, and HHO) are adopted to verify and compare the performance of the proposed algorithm. The starting search points of the algorithms selected for the comparison were the same for all the algorithms, and the simulations were performed in the same situations.

### 4.1. Benchmark Functions

The benchmark functions F1∼F8 include functions with different characteristics such as unimodal and multimodal. The unimodal function only has a strict maximum value (or minimum value) within defined upper and lower limits, which is usually used to detect the convergence speed of the algorithm. The multimodal function is a function containing multiple locally optimal solutions or global optimal solutions and is often used to detect algorithm exploration and development capabilities. The specific expression, dimension, value range, function type, and optimal value of the theoretical value of the function are shown in Table 1.

### 4.2. Algorithm Parameters

The parameter settings of each algorithm are shown in Table 2.

### 4.3. Development Environment

The software and hardware environment of the experimental platform of numerical simulation includes MatlabR2018b and Windows10, the main frequency of the machine is 2.00 GHz, and the memory is 8 GB.

In order to avoid contingency, all the algorithms are independently run in 50 comparative experiments on MatlabR2018b. The highest, lowest, average, and standard deviation of each function are calculated. The maximum number of iterations is set to 5000. The results are shown in Tables 3 and 4.

### 4.4. Experiments to Analyze the Optimality

The bold part in Tables 3 and 4 shows the optimal solution derived by the algorithm iteration under the same experimental conditions. And the optimal solutions searched by the F2 and HHO are smaller than those searched by the SCLBOA. SIBOA shows premature stagnation in F5. To sum up, the SCLBOA excels other algorithms on the benchmark function. In this experiment, just the single-modal and multimodal mathematical functions of the benchmark functions are adopted to do a model evaluation, and future experiments are suggested to incorporate composite functions.

### 4.5. Experimental Convergence Analysis

**Figure 3:**
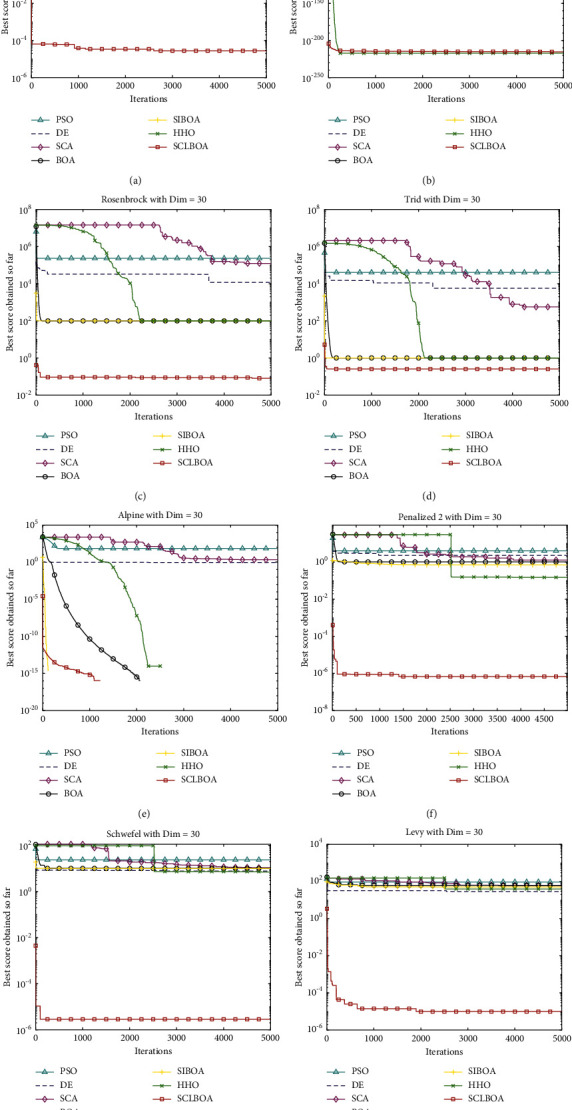
30-dimensional convergence curve of benchmark function. (a) F1. (b) F2. (c) F3. (d) F4. (e) F5. (f) F6. (g) F7. (h) F8.

**Figure 4:**
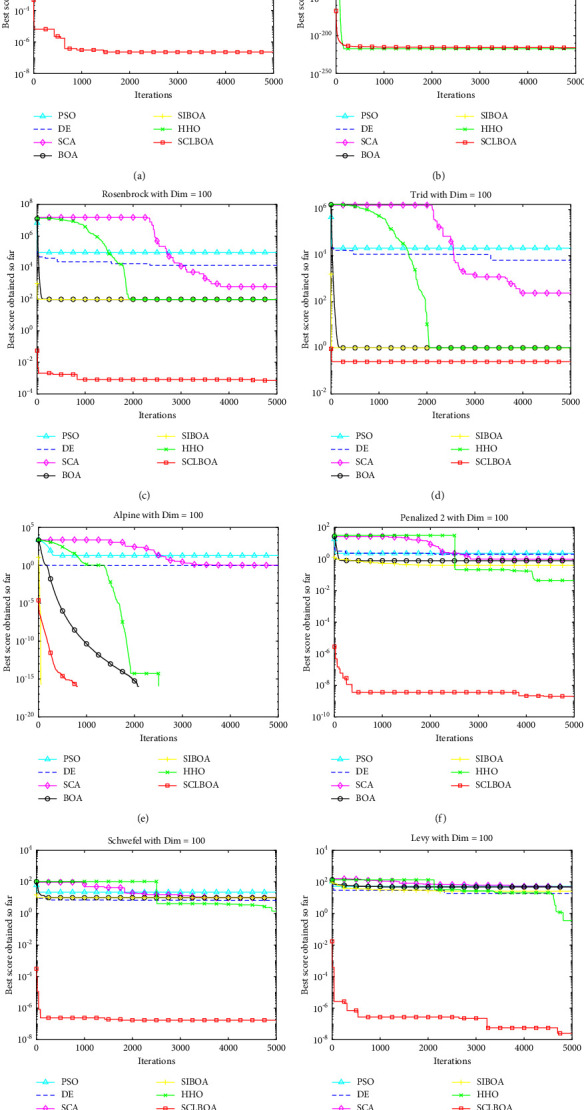
100-dimensional convergence curve of benchmark function. (a) F1. (b) F2. (c) F3. (d) F4. (e) F5. (f) F6. (g) F7. (h) F8.

The eight standard test functions in Table 1 are solved by the seven algorithms, and their fitness function value curves are shown in Figures 3 and 4. The horizontal axis represents the maximum number of iterations in the program running and the vertical axis stands for the corresponding fitness values. It can be seen from the figure that the convergence of the SCLBOA algorithm proposed in this paper is significantly better than other algorithms, which shows that the solution accuracy of the proposed algorithm has been significantly improved.

## 5. SCLBOA-BP Network Predicts Boston Housing Prices

### 5.1. BP Network

**Figure 5:**
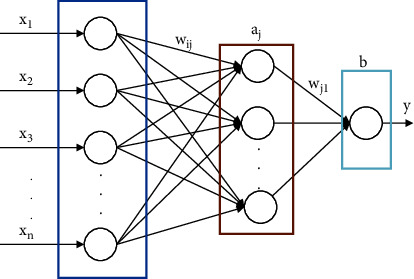
Topological structure of BP network.

BP network [33, 34] is a multilayer feedforward neural network that includes two processes which are input signal forward and error backpropagation. The structure is generally composed of an input layer, hidden layer, and output layer [35, 36]. The neuron state of each layer only affects the neuron state of the next layer. It is widely used in various prediction models. The dimensions of the input and output vectors of the training samples would determine the number of neural nodes in the input and output layers of the network, respectively. A typical BP neural network structure with only a single hidden layer and a single output is shown in Figure 5.

In Figure 5, *x*_*i*_=(*x*_1_, *x*_2_, *x*_3_, ⋯, *x*_*n*_) is a set of input vectors of the BP network; *y* is the target output value of the network; *w*_*ij*_ is the connection weight between the input layer and the hidden layer; *w*_*j*1_ is the connection weight between the hidden layer and the output layer; and *a*_*j*_ and *b* are the node thresholds of the hidden layer and output layer, respectively. If the number of hidden layer nodes is *m*, then *j* = 1,2,3,…, *m*. In the forward pass, the input signal vector *x*_*i*_ is first transmitted layer by layer from the input layer to the hidden layer and then finally to the output layer, connecting the weight vector and the threshold vector sum through each layer and calculating the corresponding activation function of each layer to get the predicted output value Y of the output layer. On the other hand, if an error occurred between the predicted value Y and the target value *y*, the error part would be transferred to the reverse layer-by-layer transmission, and the weights and thresholds of the network layers would be adjusted in the direction of reducing the error.

### 5.2. SCLBOA-BP Network

This section explains how we use the SCLBOA to train the BP network. In the SCLBOA algorithm, the position of each butterfly in the butterfly group represents a set of weights in the current iteration of the BP network, and the dimension of each butterfly represents the number of weights that play a role in the network. Meanwhile, it takes the neural network output error of a given training sample set as the fitness function of the neural network training problem. The fitness value represents the error threshold of the neural network. The smaller the error is, the better performance in the search will be. In the weight space, butterflies would move and search, which reduces the MSE of the output layer of the network. Thus, in this way, SCLBOA optimizes the search and training of the weight of the neural network to obtain a smaller output error, and in each iteration process, it would calculate and move towards a new position. The new position will be a new set of weights, and then a new MSE is obtained according to the set of weights, and the individual with the smallest MSE would be the current global optimal solution. Repeat the above process to make the predicted value of BP neural network approach the actual output value.

### 5.3. Boston Housing Price

This SCLBOA-BP network is trained and tested on the Boston house price dataset. The model's performance and predictability are evaluated. It is expected that this model can be used for in-house price estimation to improve the efficiency of real estate agents. The initial parameters of the SCLBOA-BP network are determined as follows. The group scale is 50; the maximum number of iterations Max_itera is 30; the number of input nodes is 13; the number of output nodes is 1; the number of hidden layer nodes is 13; the number of weights is 13×13+13×1 = 182; the number of thresholds is 13+1 = 14. The previous 50 sets of data are used as the test set, and the remaining 456 sets of data are used as the training set to train and test the Boston housing price prediction model. Therefore, the entire input vector is a 13 × 506 matrix. At the same time, BP, BOA-BP, and SCLBOA-BP prediction models are trained to predict the test set, compare the prediction results, and perform performance analysis.

**Figure 6:**
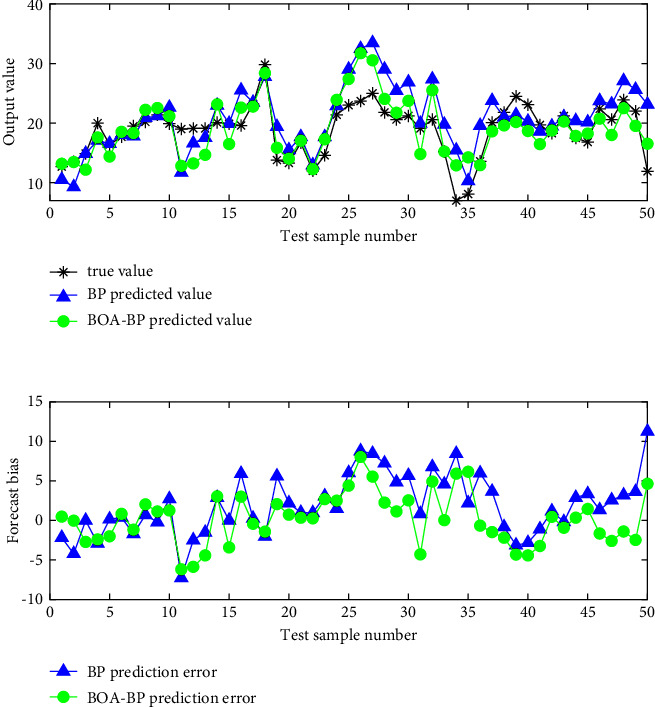
Comparison of prediction errors of BP and BOA-BP networks.

**Figure 7:**
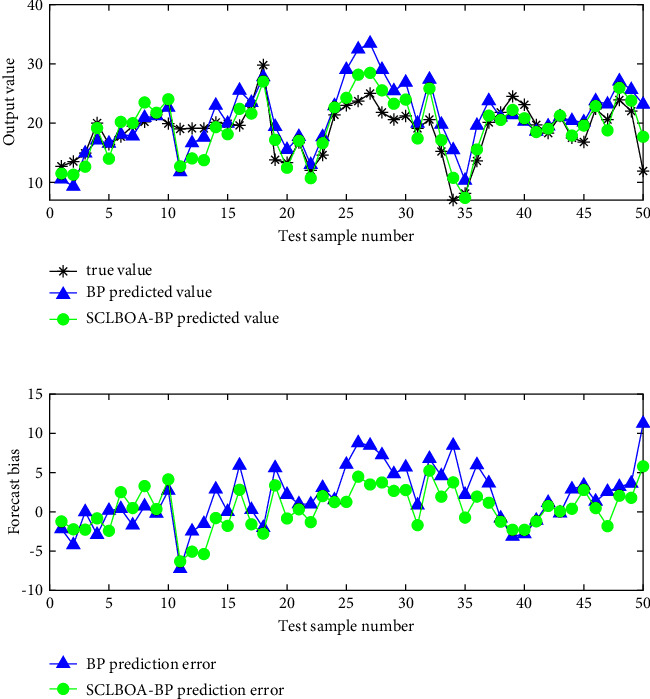
Comparison of prediction errors of BP and SCLBOA-BP networks.

Figure 6 shows the visual comparison of the BP network predicted value and the true value before and after BOA optimization. In the meantime, Figure 7 shows the visual comparison of the BP network predicted value and the true value before and after SCLBOA optimization and the error results between them. At the same time, Table 5 presents the numerical comparison of the predictive evaluation indicators, where *MAE*_*G*_, *MSE*_*G*_*, RMSE*_*G*_, and *MAPE*_*G*_ represent the average absolute error, mean square error, standard error, and average absolute percentage error in the test set, respectively, and *MSE*_*T*_ represents the mean square error in the training set.

As shown in the simulation results, the SCLBOA algorithm proposed in this paper can train the BP network to avoid the inability of finding the optimal solution in the BP training process due to the defects of the BOA itself and to avoid the algorithm from premature convergence to obtain smaller prediction error.

## 6. Discussion

The major issue in metaheuristic algorithms is being stuck in local optima. The solution search performance of the SCLBOA algorithm is tested on different functions, and the results we obtained are compared with those of other six well-established metaheuristic algorithms (i.e., PSO, DE, SCA, BOA, SIBOA, and HHO), and obtained results confirm the superiority of the proposed algorithm compared to the other metaheuristic algorithms. The real-life optimization problem, house price forecasting, is solved with the help of the newly proposed algorithm.

From the performed convergence analysis of SCLBOA, we confirm that the algorithm will guarantee convergence, but the rate of convergence is still influenced by several factors. Since the experiment is mainly to verify the effectiveness of the algorithm, the calculation time test of the algorithm is lacking, and the algorithm can be further optimized and tested in the future. Another strategy is the combination comparison of the proposed method to see if there is synergy between different strategies.

## 7. Conclusion

Butterfly optimization algorithm (BOA) has gained huge popularity among the research community and is being used to solve optimization problems in various disciplines. The SCLBOA algorithm proposed in this paper combines logistics chaotic mapping and Lévy flight mechanism based on the SIBOA. The simulation results show that the convergence speed of the algorithm is greatly improved, and the problem of the defect that is easy to fall into local optimality is significantly eliminated. Using SCLBOA as the training algorithm of the BP network, the SCLBOA-BP network is applied to train the Boston housing price prediction model, which verifies the practicability of the SCLBOA-BP network. It is noteworthy that the SCLBOA algorithm works well on the Boston house price dataset. It is recommended that future research evaluate the performance of this algorithm on real-life optimization problems.

## Figures and Tables

**Table 1:** Benchmark functions.

Name	Formula of functions	Ranges	Type	*f*_min_
Step	$F_{1}\left(x\right)=\overset{\text{dim}}{\underset{i=1}{\sum }}{\left(x_{i}+0.5\right)}^{2}$	[−10, 10]	U	0
Exponential	$F_{2}\left(x\right)=\text{exp}\left(0.5\overset{\text{dim}}{\underset{i=1}{\sum }}x_{i}\right)$	[−10, 10]	U	0
Rosenbrock	$F_{3}\left(x\right)=\overset{\text{dim}}{\underset{i=1}{\sum }}\left(100\left(x_{i+1}-x_{i}^{2}\right)+{\left(x_{i}-1\right)}^{2}\right)$	[−5, 10]	U	0
Trid	$F_{4}\left(x\right)={\left(x_{i}-1\right)}^{2}+\overset{\text{dim}}{\underset{i=1}{\sum }}i\cdot {\left(2x_{i}^{2}-x_{i-1}\right)}^{2}$	[−10, 10]	U	0
Alpine	$F_{5}\left(x\right)=\overset{\text{dim}}{\underset{i=1}{\sum }}\left|x_{i}\cdot \sin\left(x_{i}\right)+0.1x_{i}\right|$	[−10, 10]	M	0
Penalized2	$\begin{array}{c} F_{6}\left(x\right)=1/10\left\{{\text{sin}}^{2}\left(\pi x_{1}\right)+\overset{\text{dim}-1}{\underset{i=1}{\sum }}{\left(x_{i}-1\right)}^{2}\left[1+{\text{sin}}^{2}\left(3\pi x_{i+1}\right)\right]+{\left(x_{\text{dim}-1}\right)}^{2}\left[1+{\text{sin}}^{2}\left(2\pi x_{i+1}\right)\right]\right\}\\ +\overset{\text{dim}}{\underset{i=1}{\sum }}u\left(x_{i},5,100,4\right) \end{array}$	[−100, 100]	M	0
Schwefel	$F_{7}\left(x\right)=\overset{\text{dim}}{\underset{i=1}{\sum }}\left|x_{i}\cdot \sin\left(\sqrt{\left|x_{i}\right|}\right)\right|$	[−100, 100]	M	0
Lévy	$\begin{array}{c} F_{6}\left(x\right)={\text{sin}}^{2}\left(3\pi x_{i}\right)+\overset{\text{dim}-1}{\underset{i=1}{\sum }}{\left(x_{i}-2\right)}^{2}\left[1+{\text{sin}}^{2}\left(3\pi x_{i+1}\right)\right]+\\ \left|x_{\text{dim}}-1\right|\cdot \left[1+{\text{sin}}^{2}\left(2\pi x_{\text{dim}}\right)\right] \end{array}$	[−10, 10]	M	0

**Table 2:** Parameter setting of each algorithm.

Algorithm	Main parameter setting
PSO	*c*_1_=*c*_2_=1; *V*_max_=2; *V*_min_=−2; *w*=0.3
DE	*β*_max_=0.8; *β*_min_=0.2; *PCR*=0.2
SCA	*a*=2
BOA	*P*=0.8; *a*=0.1; *c*=0.01
SIBOA	*P*=0.8; *a*=0.1; *c*=0.01; *a*_max_=0.3; *a*_min_=0.01; *β*=0.5
HHO	*β*=0.5
SCLBOA	*P*=0.8; *a*=0.1; *c*=0.01; *a*_max_=0.3; *a*_min_=0.01; *β*=0.5

**Table 3:** 30-dimensional analysis of experimental results.

Function	Algorithm	Best	Worst	Avg	Std
F1	PSO	181.0466	307.1147	254.8166	46.0806
	DE	33.9266	49.3303	44.5132	6.3153
	SCA	23.9701	70.5908	41.5468	21.2888
	BOA	21.4097	23.1179	22.4268	0.6297
	SIBOA	17.6355	21.6636	19.7345	1.6576
	HHO	10.6009	17.8826	14.6023	2.7722
	SCLBOA	**2.442*E *−* *05**	**3.2606*E *−* *05**	**2.7866*E *−* *05**	**2.9876*E *−* *06**

F2	PSO	9.0741*E *−* *44	2.0754*E *−* *31	4.1508*E *−* *32	9.2814*E *−* *32
	DE	5.9582*E *−* *12	5.2214*E *−* *11	2.3922*E *−* *11	2.0591*E *−* *11
	SCA	2.6114*E *−* *98	6.7331*E *−* *95	1.5011*E *−* *95	2.9398*E *−* *95
	BOA	1.3086*E *−* *55	5.9319*E *−* *20	1.1864*E *−* *20	2.6528*E *−* *20
	SIBOA	1.4789*E *−* *74	7.6676*E *−* *53	1.5335*E *−* *53	3.4291*E *−* *53
	HHO	**7.1246*E *−* *218**	**7.1246*E *−* *218**	**7.1246*E *−* *218**	**0**
	SCLBOA	**1.6537*E *−* *216**	**6.728*E *−* *216**	**4.6404*E *−* *216**	**0**

F3	PSO	140239.9167	243377.7711	195488.8397	44764.0715
	DE	11839.8713	35188.7375	23764.3119	10714.2515
	SCA	2277.7938	290472.4339	99063.1951	118202.1576
	BOA	98.8842	98.9504	98.9070	0.0265
	SIBOA	98.8251	98.9378	98.8675	0.0422
	HHO	98.5189	98.6574	98.5958	0.0521
	SCLBOA	**0.0571**	**0.0837**	**0.0749**	**0.0103**

F4	PSO	35632.7692	67202.0223	46545.5662	12160.8160
	DE	3852.1301	13770.3416	8823.3375	4093.7086
	SCA	330.1615	39322.9625	9019.2035	17021.1627
	BOA	0.9985	0.9993	0.9989	0.0003
	SIBOA	0.9950	0.9979	0.9965	0.0011
	HHO	0.9989	1.0000	0.9997	0.0005
	SCLBOA	**0.2526**	**0.2533**	**0.2528**	**0.0003**

F5	PSO	52.5303	93.1237	75.2543	15.8363
	DE	0.9282	0.9851	0.9639	0.0228
	SCA	2.0739	11.6098	5.3512	3.8515
	BOA	**0**	**0**	**0**	**0**
	SIBOA	**0**	**0**	**0**	**0**
	HHO	**0**	**0**	**0**	**0**
	SCLBOA	**0**	**0**	**0**	**0**

F6	PSO	2.6915	4.3265	3.6479	0.7616
	DE	1.5783	2.5096	2.1159	0.3542
	SCA	1.1047	1.5366	1.3326	0.1570
	BOA	0.8182	1.0525	0.9715	0.0915
	SIBOA	0.5146	0.7051	0.6039	0.0789
	HHO	0.1357	0.6058	0.2748	0.1915
	SCLBOA	**3.0132*E *−* *07**	**7.0776*E *−* *06**	**1.8989*E *−* *06**	**2.9017*E *−* *06**

F7	PSO	23.6924	30.0171	26.6267	2.6927
	DE	5.7179	8.8331	7.5446	1.2827
	SCA	9.8780	10.7831	10.2416	0.3511
	BOA	9.9811	9.9942	9.9879	0.0049
	SIBOA	9.9762	9.9935	9.9837	0.0064
	HHO	2.9914	7.4718	5.4866	2.2858
	SCLBOA	**1.995*E *−* *07**	**6.4009*E *−* *06**	**3.6767*E *−* *06**	**2.4289*E *−* *06**

F8	PSO	65.2500	96.4219	80.7352	13.9351
	DE	22.6352	30.5916	27.4761	3.0171
	SCA	55.6301	64.542	60.6603	3.9797
	BOA	47.4816	63.2023	58.2287	6.3632
	SIBOA	28.5665	53.8007	44.8094	9.8536
	HHO	1.1811	39.8297	9.7527	16.8752
	SCLBOA	**9.8249*E *−* *06**	**2.8067*E *−* *05**	**1.8968*E *−* *05**	**7.7381*E *−* *06**

**Table 4:** 100-dimensional analysis of experimental results.

Function	Algorithm	Best	Worst	Avg	Std
F1	PSO	166.3619	205.7117	184.3892	16.3906
	DE	40.1283	48.2946	43.8668	3.3597
	SCA	21.3722	32.4426	24.5616	4.5361
	BOA	20.9834	22.6447	22.0810	0.7283
	SIBOA	10.9430	16.1625	14.2100	2.3283
	HHO	4.2899	7.8830	6.4240	1.4162
	SCLBOA	**6.5225*E *−* *08**	**3.2177*E *−* *07**	**2.2888*E *−* *07**	**9.7767*E *−* *08**

F2	PSO	3.8062*E *−* *60	5.3637*E *−* *37	1.0727*E *−* *37	2.3987*E *−* *37
	DE	3.4098*E *−* *14	6.842*E *−* *12	3.3576*E *−* *12	2.9254*E *−* *12
	SCA	1.0787*E *−* *110	2.316*E *−* *100	6.051*E *−* *101	1.0042*E *−* *100
	BOA	1.8496*E *−* *72	2.0661*E *−* *36	4.1322*E *−* *37	9.2399*E *−* *37
	SIBOA	9.9255*E *−* *93	6.7142*E *−* *79	4.0286*E *−* *79	3.6774*E *−* *79
	HHO	**7.1246*E *−* *218**	**7.1246*E *−* *218**	**7.1246*E *−* *218**	**0**
	SCLBOA	**1.2383*E *−* *216**	**2.056*E *−* *216**	**1.4769*E *−* *216**	**0**

F3	PSO	90692.9315	140515.2230	118456.9363	20760.1363
	DE	9871.3375	20299.4107	13178.9125	4327.3757
	SCA	620.2451	55904.5248	22780.9838	23313.5681
	BOA	98.8360	98.9177	98.8696	0.0328
	SIBOA	98.5826	98.7606	98.6448	0.0695
	HHO	98.2441	98.2830	98.2658	0.0173
	SCLBOA	**0.0006**	**0.0008**	**0.0007**	**7.2565*E *−* *05**

F4	PSO	21127.2238	32223.9219	26148.6597	4345.0129
	DE	6282.9754	9259.7245	7784.3796	1258.2453
	SCA	238.3159	4856.4503	1888.1436	1896.3551
	BOA	0.9981	0.9988	0.9984	0.0003
	SIBOA	0.9866	0.9919	0.9887	0.0021
	HHO	0.9985	1.0000	0.9997	0.0007
	SCLBOA	**0.2499**	**0.2500**	**0.2500**	**2.7724*E *−* *05**

F5	PSO	19.1422	25.6965	22.2168	2.3686
	DE	0.8677	0.9558	0.9204	0.0328
	SCA	0.3738	2.5592	1.2125	0.8094
	BOA	**0**	**0**	**0**	**0**
	SIBOA	**0**	**0**	**0**	**0**
	HHO	0	0	0	0
	SCLBOA	0	0	0	0

F6	PSO	2.1051	2.5453	2.3114	0.2214
	DE	1.5428	2.0325	1.7948	0.2451
	SCA	0.9820	1.2824	1.0960	0.1627
	BOA	0.7780	0.9038	0.8356	0.0635
	SIBOA	0.2969	0.3963	0.3317	0.0560
	HHO	0.0313	0.1258	0.0673	0.0512
	SCLBOA	**1.9045*E *−* *09**	**2.2081*E *−* *09**	**2.0529*E *−* *09**	**1.5192*E *−* *10**

F7	PSO	16.3137	22.2838	19.1493	2.9963
	DE	5.0175	6.9523	6.2227	1.0514
	SCA	9.7129	10.6423	10.2291	0.4732
	BOA	9.9769	9.9950	9.9841	0.0096
	SIBOA	9.9536	9.9616	9.9573	0.0040
	HHO	0.8146	2.4986	1.5703	0.8552
	SCLBOA	**1.1533*E *−* *08**	**1.7277*E *−* *07**	**9.3842*E *−* *08**	**8.067*E *−* *08**

F8	PSO	51.8956	63.0998	58.4043	5.8180
	DE	18.7263	27.7898	22.0143	5.0177
	SCA	49.7574	63.8980	55.1068	7.6729
	BOA	46.4013	50.9537	48.1928	2.4261
	SIBOA	16.8091	25.6142	21.6965	4.4820
	HHO	0.3503	18.5150	6.7313	10.2167
	SCLBOA	**2.5586*E *−* *08**	**1.2642*E *−* *07**	**8.7767*E *−* *08**	**5.4379*E *−* *08**

**Table 5:** Error results.

Predictive evaluation index	BP	BOA-BP	SCLBOA-BP
*MAE*_*G*_	3.252	2.5572	2.2617
*MSE*_*G*_	17.6617	10.207	7.4199
*RMSE*_*G*_	4.2026	3.1948	2.724
*MAPEE*_*G*_	19.6249%	15.0638%	11.0334%
*MSE*_*G*_	0.011434		

## Data Availability

The Boston housing dataset can be found in the following website: https://www.kaggle.com/schirmerchad/bostonhoustingmlnd.
